# RETRACTION: Role of Hydrocolloid Dressing in Preventing Face Pressure Ulcers From Non‐Invasive Ventilation and Facilitating Postoperative Wound Healing in Maxillofacial Surgery: A Meta‐Analysis

**DOI:** 10.1111/iwj.70586

**Published:** 2025-04-18

## Abstract

**RETRACTION:** WangJ. , GaoQ. , FengX. , and ChenY. , “Role of Hydrocolloid Dressing in Preventing Face Pressure Ulcers From Non‐Invasive Ventilation and Facilitating Postoperative Wound Healing in Maxillofacial Surgery: A Meta‐Analysis,” International Wound Journal 21, no. 3 (2024): e14780, 10.1111/iwj.14780.38385780 PMC10883250

The above article, published online on 22 February 2024, in Wiley Online Library (http://onlinelibrary.wiley.com/), has been retracted by agreement between the journal Editor in Chief, Professor Keith Harding; and John Wiley & Sons Ltd. Following an investigation by the publisher, all parties have concluded that this article was accepted solely on the basis of a compromised peer review process. The editors have therefore decided to retract the article. The authors did not respond to our notice regarding the retraction.

**RETRACTION:** J. Wang , Q. Gao , X. Feng , and Y. Chen , “Role of Hydrocolloid Dressing in Preventing Face Pressure Ulcers From Non‐Invasive Ventilation and Facilitating Postoperative Wound Healing in Maxillofacial Surgery: A Meta‐Analysis,” International Wound Journal 21, no. 3 (2024): e14780, 10.1111/iwj.14780.38385780 PMC10883250

The above article, published online on 22 February 2024, in Wiley Online Library (http://onlinelibrary.wiley.com/), has been retracted by agreement between the journal Editor in Chief, Professor Keith Harding; and John Wiley & Sons Ltd. Following an investigation by the publisher, all parties have concluded that this article was accepted solely on the basis of a compromised peer review process. The editors have therefore decided to retract the article. The authors did not respond to our notice regarding the retraction.

